# A novel, optical, on-line bacteria sensor for monitoring drinking water quality

**DOI:** 10.1038/srep23935

**Published:** 2016-04-04

**Authors:** Bo Højris, Sarah Christine Boesgaard Christensen, Hans-Jørgen Albrechtsen, Christian Smith, Mathis Dahlqvist

**Affiliations:** 1 GRUNDFOS Holding A/S, Poul Due Jensens Vej 7, DK-8850 Bjerringbro, Denmark; 2 HOFOR A/S, Ørestads Boulevard 35, DK-2300 København S, Denmark; 3 Technical University of Denmark, Department of Environmental Engineering, Miljøvej, Building 115, 2800 Kgs. Lyngby, Denmark

## Abstract

Today, microbial drinking water quality is monitored through either time-consuming laboratory methods or indirect on-line measurements. Results are thus either delayed or insufficient to support proactive action. A novel, optical, on-line bacteria sensor with a 10-minute time resolution has been developed. The sensor is based on 3D image recognition, and the obtained pictures are analyzed with algorithms considering 59 quantified image parameters. The sensor counts individual suspended particles and classifies them as either bacteria or abiotic particles. The technology is capable of distinguishing and quantifying bacteria and particles in pure and mixed suspensions, and the quantification correlates with total bacterial counts. Several field applications have demonstrated that the technology can monitor changes in the concentration of bacteria, and is thus well suited for rapid detection of critical conditions such as pollution events in drinking water.

One of the major challenges in ensuring safe drinking water is the difference between the time it takes to produce, distribute, and consume the water, and the time it takes to investigate whether it is safe to drink^1^ it. In drinking water some of the major health risks are constituted by microorganisms^2^,^3^,^4^,^5^ either coming from the water source, entering storage or distribution systems unintendedly or growing in the water. Unfortunately, by the time routine microbial analysis reveals a possible bacterial pollution, the investigated water has often already been distributed and consumed.

Water utilities are required to verify the water quality on a regular basis, applying standard methods at predetermined sampling frequencies. These methods are typically growth-based, laborious and time-consuming, giving answers one to three days later^6^ and merely providing point information without insight into temporal development. Further, application of heterotrophic plate count methods only reveal a fraction of the total population present in drinking water as they do not include viable, but non-culturable bacteria^7^,^8^,^9^.

Automating existing technology for on-line detection of bacteria^10^,^11^, e.g. flow cytometry^12^,^13^, or indirect indicators of bacterial activity, such as ATP^14^, have been given much attention over the past years. A great deal of effort has also been put into the development of sensors that sense bacteria by direct contact with the sensor surface^15^,^16^,^17^. Unfortunately, these solutions are either complicated to operate, require addition of chemicals, daily or weekly maintenance, or are too expensive to be deployed throughout distribution networks. The contact type sensors further encompass the probability of a bacterium actually touching the sensor surface. Considering the relatively low concentration of bacteria in drinking water, this probability may be very low.

Since many parameters in drinking water systems may vary significantly, spatially and timely, it is likely that routine monitoring with lab sampling will fail to catch short-term pollutions^18^. Consequently, major utilities often increase the number of analyses beyond the requirements and supplement their data with on-line measurements of turbidity, conductivity, etc.^19^,^20^,^21^ Since such parameters respond to more than just bacterial content, they are likely to show false positives as well as false negatives in terms of microbiological pollution detection.

Conclusively, the delay and limitations associated with current growth-based methods and the missing specificity of current on-line methods make it practically impossible to proactively react on contamination events in today’s drinking water distribution systems. What seems to be missing in this technology gap is a compromise between the two extremes: A sensor that may have a longer response time than the indirect sensors (pH, conductivity, etc.) and may be far less specific than the laboratory-based methods, but instead provides valuable information on the dynamics of bacteria concentrations in general. For such a sensor to be applicable in remote locations, e.g. throughout a drinking water distribution network, it should need as little maintenance as possible, should not require chemical supplies, and should not create hazardous waste.

In this paper, we present a rapid, chemical-free method for on-line monitoring of non-specific bacteria in water with a 10-minute time resolution, based on 3D scanning by a moving digital microscope. We aim to prove the sensor concept, demonstrating its applicability to distinguish between microbial and abiotic particles, and detect variations so fast that it enables proactive actions to potential pollution events, thus providing a new tool for risk management in drinking water applications. The ability of the method to quantify particles, measure their size and eccentricity, and classify them as either bacteria or abiotic particles, has been proved through laboratory tests. The applicability and robustness of the method in on-line monitoring have been demonstrated through field tests. Various drinking water systems have been monitored by the method revealing both stable base lines and responses to various events.

## Results

### Measuring principle

**Figure 1:**
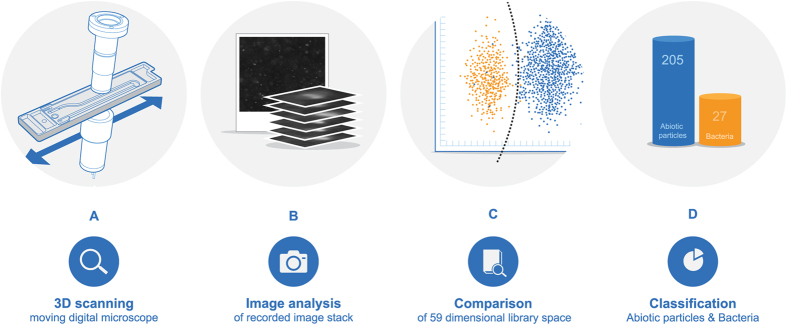
The various steps in determining the concentration of bacteria and abiotic particles. (**A**) Schematic of flow cell, light source, lens, and camera. (**B**) Image stack of a particle coming into focus and out again as the tilted image plane moves across it. (**C**) Extraction of parameters from recorded image stacks and comparison to library data. (**D**) Classification of particles in “Bacteria” and “Abiotic particles”.

The developed sensor consists of: 1) an optical flow-cell holding the water sample during analysis, 2) a dark field imaging setup with a light-emitting diode (LED) light source, a magnification lens, and a complementary metal-oxide semiconductor (CMOS)-based camera arrangement (Fig. 1A), and 3) an image analysis system to identify and classify individual particles.

The water sample is led through the flow-cell by the pressure of the water source or by a peristaltic inlet pump. Initiating each measurement, the water sample is sealed inside the flow cell, designed to minimize internal circulation and thus fix the particles spatially. The sample is stagnant in the flow-cell while scanned along the length of the flow-cell by the tilted imaging setup, causing the acquired images to span a fixed volume of 6 μl (Fig. 1A). After sampling and image acquisition, the cell is flushed for one minute by the source water, preparing it for the next sample.

The camera is only moved a small fraction of the image length (2.3%) between individual images, causing each particle to be recorded on up to 40 images. Due to the tilting of the acquisition setup, each particle may be out of focus on the first images, in focus on the middle images, and out of focus again on the later images^22^ (Fig. 1B). Besides information about the particle in focus, information about the diffraction of light by the particular particle is also recorded, allowing for classification of each particle into specific particle classes, i.e. bacteria and abiotic particles.

Our newly developed software divides the images into segments containing individual particles, which are subsequently gathered in image stacks of 10 to 40 images. From each image stack, 59 optical parameters related to the nature of the original particle is extracted. Some spatial parameters are linked to a thresholded image of the particle in focus, e.g. area, length, perimeter, eccentricity, convexity, and binary moments. Other parameters are linked to the grayscale image, e.g. contrast, light-scattering properties, absorption, moments, granularity, and features extracted from Fourier-transformed image spaces^23^. The size feature, dividing the identified particles into 20 size bins ranging from <0.62 μm to >9.5 μm, can be used as an additional indicator of deviations from the normal baseline of particle size distribution in the particular water matrix.

The results, such as concentrations of bacteria and abiotic particles, particle eccentricities or sizes, are transferred wirelessly to a data server that handles and distributes the data to the users. At server level, further algorithms can be applied to set off alarms based on e.g. levels of bacteria or certain patterns in the development of the bacteria concentration.

### Particle classification

To distinguish between different types of particles, e.g. bacteria versus abiotic particles, a software library was established from scanning suspensions of known particles from each group i.e. bacteria and abiotic particles. The library included morphologically different bacterial shapes, such as rods, curved rods and cocci, as well as various abiotic particles relevant to drinking water, e.g. humic acid, clay, and iron oxides. Each suspension scan included 59 measured parameter values to characterize the specific particle types.

Neural networks were used to establish a boundary between bacteria and abiotic particles in the 59-dimensional space spanned by these parameters. The result was an algorithm capable of distinguishing between bacteria and abiotic particles based on image acquisition (Fig. 1C). Illustrating the 59-dimensional space is not graphically possible, which is why the graph in Fig. 1C is a mere projection onto two dimensions.

By comparing each particle recorded in an unknown sample with the library, the individual particles may be classified as either a “bacteria” or an “abiotic particle” (Fig. 1D).

### Handling fouling of the optical windows

The background of scattered light in the recorded image is measured as an indication of fouling on the wetted surfaces of the flow cell. Biotic or abiotic fouling on the flow-cell window diffracts the light and increases the background level of the recorded dark-field image. By monitoring this level and setting limits for flow-cell exchange based on these levels, the influence of fouling on the measured concentrations is confined to an acceptable and negligible level. The sensor tracks the fouling level of the flow-cell with the same time resolution as the particle measurements. When the flow-cell needs to be replaced, the sensor alerts the user.

If for example an air bubble or a significant spot of fouling should affect a small fraction of the analyzed volume, the sensor rejects this volume as bad volume and corrects the analyzed volume accordingly.

### Proof of concept

The concept of detecting, measuring, and classifying particles was evaluated for: 1) accuracy of classification measure in pure and mixed suspensions, 2) accuracy of size measure, 3) linearity of concentration measure.

**Figure 2:**
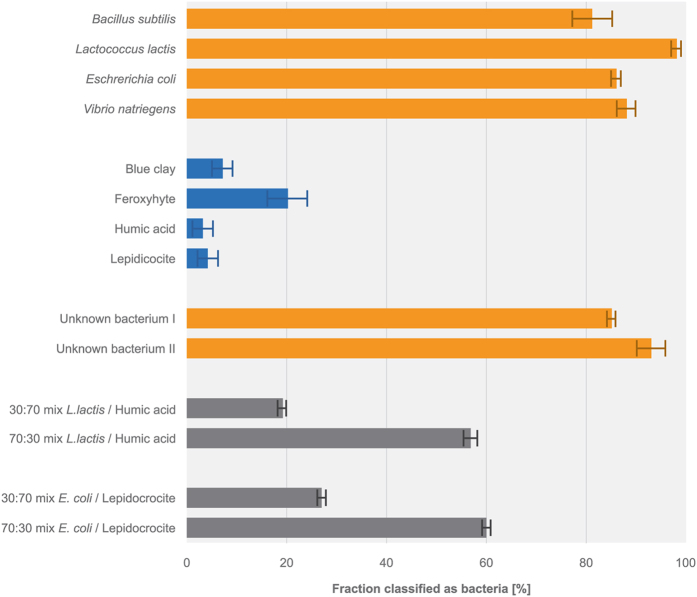
Ability of the sensor to distinguish between bacteria and abiotic particles in suspensions shown as the fraction classified as bacteria. The suspension included model particles in pure suspensions (orange: bacteria, blue: abiotic particles) and mixtures of bacteria and abiotic particles (grey). Mixed suspensions would ideally be reported as 30 and 70% bacteria if they were classified 100% correctly. Error bars represent standard deviations.

To evaluate the ability of the sensor to classify particles correctly, we used the sensor to analyze a series of monotype suspensions of known abiotic particles and bacteria, including pure cultures of known bacteria, unknown bacteria isolated from drinking water and abiotic particles. The classification accuracy (Fig. 2) was determined as the percentage of identified particles assigned to the correct class, e.g. the percentage of abiotic particles (96 ± 2%, s.d.) reported for a suspension of lepidocrocite or the percentage of bacteria (88 ± 2%, s.d.) reported for a suspension of *Vibrio natriegens*. For monotype suspensions, the classification accuracy was 90 ± 7% (s.d.).

Mixed suspensions of *Lactococcus lactis* and humic acid and mixed suspensions of *E. coli* and lepidocrocite were prepared in ratios of 30/70 and 70/30. When analyzed by the sensor, the ratio between classified bacteria and abiotic particles was 19/81 and 57/43 in the ‘*L. lactis* and humic acid’ suspension and 27/73 and 60/40 in the ‘*E. coli* and lepidocrocite’ suspension (Fig. 2). For mixed-type suspensions, the average classification accuracy was 78 ± 14% (s.d.).

**Figure 3:**
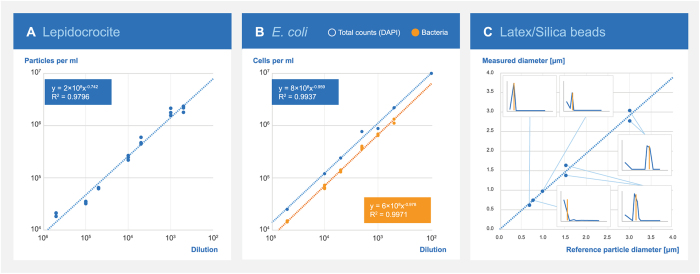
Linearity of the sensor signal and size measures. (**A**) Linearity of the sensor signal for abiotic particles in tenfold dilutions of lepidocrocite. (**B**) Linearity of the sensor signal for bacteria in tenfold dilutions of *E. coli* as well as total bacterial counts. (**C**) Accuracy of particle size measured by the sensor, standard diameter refers to certified sizes of standard particles used. Inserts indicate the measured size distribution (by number of particles) with indication of the actual size (vertical orange lines). For peaks split between two size bins, both bin sizes are plotted.

To address the linearity of the sensor signal, a dilution series covering three decades of suspensions of 1) lepidocrocite particles (Fig. 3A) and 2) *E. coli* (Fig. 3B) were analyzed by the sensor. The analysis revealed linear regression coefficients of determination (*R*^*2*^) of 0.9796 and 0.9971, respectably. For comparison, linear regression between dilution factors of the *E. coli* suspension and total bacterial counts by DAPI staining gave an *R*^*2*^ of 0.9889.

**Figure 4:**
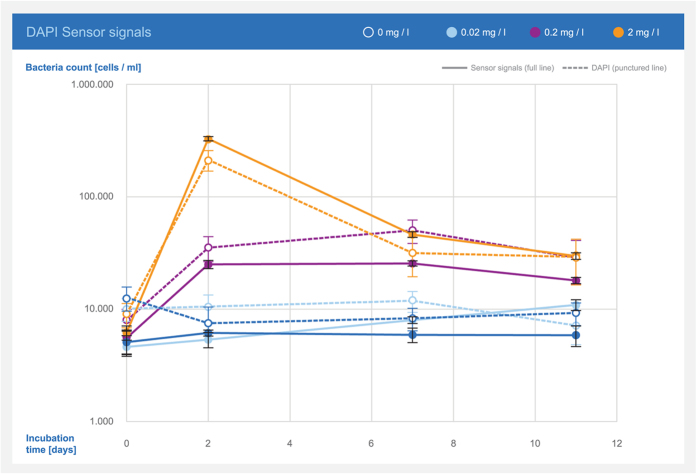
Development of total bacterial counts in drinking water with added yeast extract as reported by the sensor and measured through DAPI staining. Error bars represent standard deviations.

To further investigate the correlation between sensor response and total counts, the growth of bacteria in tap water with different nutrient additions was followed by both methods (Fig. 4). The results showed stagnant bacterial numbers for tap water with no addition and with 0.02 mg/l yeast extract. When 0.2 and 2 mg/l yeast extract was added, the results showed increasing bacterial numbers with a maximum after 2–7 days (0.2 mg/l) and 2 days (2 mg/l) followed by a decrease in the number of bacteria. The sensor response was similar to the concentrations obtained by DAPI staining (*R*^*2*^ 0.9727).

The size feature of the algorithm, dividing the identified particles into 20 size bins ranging from <0.62 μm to >9.5 μm, was verified by analyzing suspensions of certified monodisperse polystyrene beads of 0.771, 0.99, 1.53, and 3.004 μm in diameter. These were chosen to represent bacteria sizes expected to occur in drinking water. Particle sizes reported by the sensor decreased in the correct size bin, approximately within 5% of the correct diameter, or in the adjacent bin up to 10% off the correct value (Fig. 3C). The sensor reported silica beads of 0.69 μm in diameter in the size bin of 0–0.62 μm. Larger particles with a relatively high density may be expected to precipitate during the image acquisition, and the concentration of such particles may thus be underestimated by the sensor.

Based on the relatively small volume measured inside the flow cell, the theoretical detection limit (a single particle detected) is 1.6 × 10^2^ particles/ml, which is also the theoretical resolution provided the entire flow-cell volume is included in the measurement. Sampling from a water source with such a low concentration of particles would be associated with a high degree of uncertainty due to sample inhomogeneity. The sensor is designed to operate in drinking water, which seldom contains less than a few thousand particles/bacteria per ml, according to our field test experiences. Depending on the nature of the particles and how much background scattering they cause, the upper limit has proved to be around 1–5 × 10^6^ particles/ml. Above this concentration, small particles are not detected due to the high background scattering, and the sensor thus underestimates the concentrations.

### Field-testing

**Figure 5:**
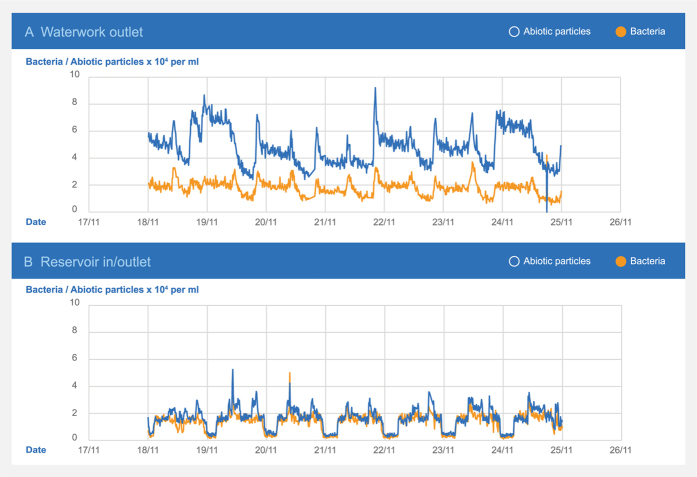
Dynamics of the concentration of bacteria and abiotic particles at two locations in a large Danish drinking water distribution system measured by the sensor. At the waterworks outlet, the concentration of abiotic particles was approximately three times higher than the bacteria concentration. At the downstream reservoir, the concentration of bacteria and abiotic particles were similar and significantly lower as compared to the waterworks. The substantial variations at the reservoir were caused by the flow direction, with highest concentrations in the water coming from the reservoir.

The sensor was investigated in various drinking water systems, primarily Danish and French. In a Danish non-chlorinated distribution system, one sensor was installed at a waterworks and one at a downstream reservoir (Fig. 5). A 9-km-long pipeline connected the waterworks and the reservoir. The water had an average transport time of 32 hours with an average flow velocity of 0.08 m/sec. The abiotic particle concentration was substantially lower in the reservoir water than in the waterworks effluent, probably due to sedimentation during the transport as a result of the very low flow velocity. The bacteria concentration did not change accordingly, and the sensor thus clearly detected different behavior of abiotic particles and bacteria.

**Figure 6:**
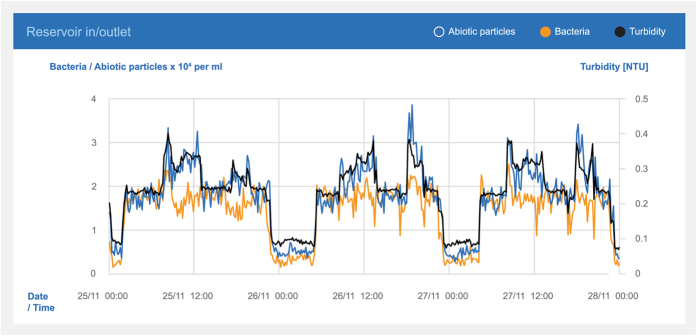
Turbidity, bacteria, and abiotic particles measured at the inlet/outlet of a Danish clean water reservoir. Note the distinct difference between the water entering (around 5 × 10^3^ cells/ml) and leaving (2–4 × 10^4^ cells/ml) the reservoir.

The pipe was used as inlet to as well as outlet from the reservoir. The sensor recorded alternating water qualities reflecting whether the water came from the pipeline or the reservoir. The turbidity and the measured concentration of the abiotic particles showed similar behavior (R^2^ 0.92). The measurement of the bacteria (Fig. 6) differed clearly from these patterns (R^2^ 0.78) and turbidity were thus less able to detect the changes in the bacteria concentration.

**Figure 7:**
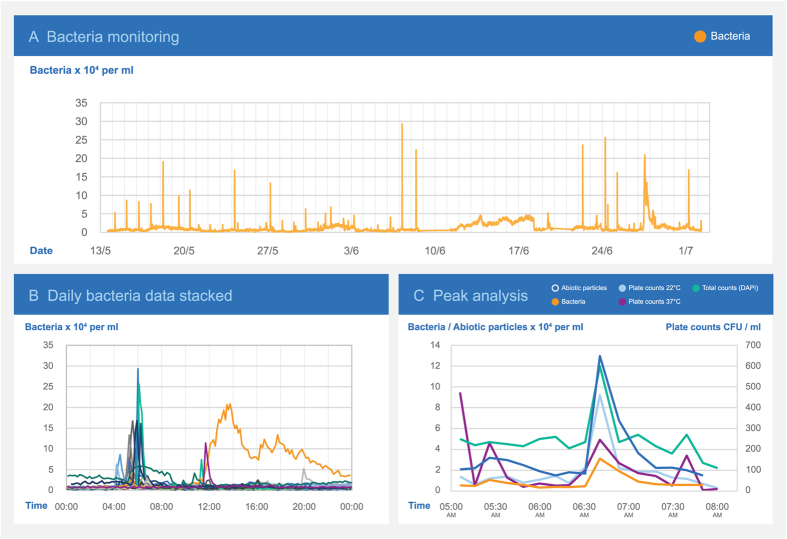
Water quality monitoring at a Danish cattle slaughterhouse. (**A**) Part of the 11 weeks monitoring campaign showing bacteria peaks occurring daily. (**B**) Overlaying daily bacteria curves for normal workdays show a daily peak occurring in the early morning. One day also showed a gradually increasing bacterial concentration. (**C**) Applying the developed bacteria sensor, total bacteria counts, and plate counts. Peaks for plate counts at 22 °C and total counts co-occurred with the peaks measured by the sensor. However, plate counts at 37 °C showed peaks that did not correlate with other parameters.

The sensor was also installed at a Danish cattle slaughterhouse, where the water quality did not meet guideline values (based on routine analyses of plate count numbers). A notice to boil the water prior to consumption had been issued. A sensor was installed to monitor the water inlet to the slaughterhouse by sampling every ten minutes for 11 weeks. The sampling revealed a daily peak in the concentration of both bacteria and abiotic particles (Fig. 7A). Overlaying individual days of data showed the peak to occur in the morning between 5:30 and 7:30 in the morning (Fig. 7B). During the peak, the sensor detected an increase in the bacteria concentration from 1–4 × 10^4^ to 2–5 × 10^5^ cells/ml and an increase in the concentration of abiotic particles from 3–5 × 10^4^ to 3–8 × 10^5^ particles/ml.

During a sampling campaign around the estimated time of the peak, grab samples of the water was collected before each sensor measurement and analyzed for total counts and plate counts at 22 and 37 °C. The bacteria and abiotic particles recorded by the sensor correlated well with the water quality analyses at the time of the peak (Fig. 7C). From DAPI staining of non-homogenized samples, it was noted that part of the bacteria within the samples were clustered together in groups or pieces of biofilm, which could explain why the total numbers in the homogenized sample used for total counts were 3–4 times higher than the numbers quantified by the sensor.

It was discovered that the reason why the microbial water quality guidelines were exceeded was a combination of stagnant water in an inappropriate piping layout upstream from the sensor location and the onset of the morning cleaning. The lack of flow during the night prevented the sensor from picking up the slow increase in bacteria concentration that was expected to have occurred upstream.

## Discussion

The development of the presented sensor focused on the recognition of particles based on shape and pattern of light diffraction. From a size distribution point of view, emphasis was put into classifying particles in the range of 500 nm to a few micrometers according to the size of most bacteria^24^. The library used to build the classification algorithm was thus dominated by particles in this size range. From field test data, we have experienced that particles above 5 μm are scarce, and particles above 10 μm hardly occur within the sample volume. Particle size distribution in Danish drinking water measured by Coulter Counter (data not shown) typically showed that less than 0.1% of the particles were larger than 5 μm. Thus, the influence of particle sedimentation can be ignored for the majority of particles.

In most optical imaging systems, a spatial resolution limit is reached around 1 μm. The imaging system presented uses a 505 nm light source and a lens with a numerical aperture of 0.2. The Abbe diffraction limit for such a system is 1.26 μm^25^, which is well above the bacteria sizes characterized. Due to the added information about how the particles behave over time and in and out of focus, it is possible to characterize objects below the resolution limit of the imaging system. Particles larger than 3.0 μm were not investigated. However, the accuracy is expected to be at least as good as for the smaller sizes, due to the higher number of pixels covered.

Fouling of the flow-cell is unavoidable, as it is the case with all optical sensor windows. Since the flow-cell is easy to replace, the fouling of the optical window can essentially be kept at a very low level where it does not influence the measurements. In drinking water, the time between flow-cell changes may be several months, whereas in water with a higher fouling potential, the service intervals may be significantly shorter.

The optical on-line bacteria sensor revealed bacteria concentrations which correlated well with total bacterial counts. Such correlation was expected as similar requirements apply for a cell to be identified by both methods; it has to be intact, but not necessarily alive and active. Dead and inactive bacteria are detected by both methods if they are intact. Once the cell is ruptured by e.g. treatment with ozone, DNA will leak out of the cell, and it will no longer be detected by DAPI staining. Cell debris will most likely not be classified as “bacteria”, but instead as “abiotic particles” by the sensor. The classification shift for a decaying cell has not been fully investigated yet. The immediate and long-term effect of various disinfectants on the detection of bacteria is thus not clearly understood. However, it is obvious that the effect of UV treatment and biostatic treatment with low residual chlorine concentrations, which does not change the morphology of the cells significantly, will not be immediately visible by this technique, as it will include both active and inactive cells. We have observed that total bacterial counts by DAPI staining yields a higher number compared to the sensor, depending on the degree of aggregation among the bacteria. The main reason is that the microscope operator counting stained fluorescent cells is able to distinguish individual aggregated cells, whereas the sensor detects such cells as a single particle.

There were no simple correlations between the sensor bacteria counts and plate counts. Because the plate count method only reveals a fraction of the current population, and since this fraction may vary concurrently with the change in the population, e.g. during a pollution event, a simple correlation was not expected. Similar observations have been reported for total bacterial counts^26^.

By the ability of closely monitor trends in the total number of bacteria and other particles in drinking water with a frequency of 10 minutes, we have demonstrated that it is possible to detect slow or sudden changes that may influence the water quality, and further determine if such changes are primarily of biotic or abiotic nature. Information on the particle size distribution, which is also provided by the sensor, may serve as further indicators of pollution^27^,^28^. Such pollution indications may be used to trigger auto-sampling at opportune moments followed by detailed analyses of the event. In such cases, the combination of sampling at the right time and analyzing for relevant parameters using fast molecular microbiological methods^29^ may prove to be a powerful tool for source tracking as well as for obtaining specific information on the pollution event.

Flow cytometry has lately been demonstrated to be applicable for online measurements^12^,^13^. Flow cytometry has a measurement range (1 × 10^3^–1 × 10^6^ cells/ml) similar to the operating range presented in this paper (1.6 × 10^2^–1–5 × 10^6^ cells/ml) and an error of less than 5%. The method requires chemical staining solutions and may thus be limited to locations where these can be stored and exchanged regularly. Chemical reactants are also required for the online measurements of ATP^14^ as an indirect indicator of bacteria presence. Turbidity, as implemented in many drinking water distribution systems, may give a good indication of abiotic particles, but it is unlikely to correlate with total bacterial counts^30^.

The optical sensor has proven to be stable for several months during field-testing in waterworks environments. The most critical parameter determining the integrity of the measured concentrations and classification has been fouling of the optical flow cell. In Danish non-chlorinated drinking water, flow-cells were replaced after approximately two months, whereas in the French, chlorinated drinking water flow-cells lasted around four months.

## Conclusion

We have presented a novel optical sensor with a 10-minute resolution, capable of counting particles in water and classifying them as either bacteria or abiotic particles with a certainty of 90 ± 7% for monotype suspensions and 78 ± 14% for mixed-type suspensions. The results from the sensor correlated with total bacterial counts by DAPI staining and epi-fluorescence microscopy and responded linearly to serial dilutions. The sensor operates within a range of 1.6 × 10^2^–1–5 × 10^6^ particles/ml, the lower theoretical level corresponding to the detection of a single particle, and reports correct particle sizes in the range of 0.77–3 μm. Smaller particles or bacteria may not be detected or classified correctly.

During field tests, the sensor has been stable for up to four months without maintenance. In non-chlorinated waters, maintenance in terms of flow-cell changes are required at intervals of around two months. In other waters with high fouling potentials, higher maintenance frequencies are required. In a field test, the sensor was able to pinpoint the time of a daily reoccurring bacterial peak event, allowing for targeted sampling and detailed verification of the cause.

Analyses throughout a drinking water distribution system demonstrated that the optical sensor can monitor the different dynamics of bacteria and abiotic particle levels in drinking water and may serve as an early warning for drinking water pollution. Used as such, the sensor enables proactive responses to pollution events such as change of treatment scheme or redirection of water flows to avoid polluted water to reach consumers.

## Materials and Methods

### Laboratory sensor set-up

For laboratory verification, three sensors were modified by disabling automatic sample intake and rinsing. This allowed for manual preparation of the flow cell, manual flushing, and manual introduction of the individual samples.

### Preparation of glassware and flow cell

All glassware were washed, autoclaved at 121 °C and 15 psi for 20 minutes, rinsed three times with filter sterilized (0.22 μm polyethersulfone (PES)) tap water, and finally rinsed once with 0.22 μm filtered 70% ethanol before left to dry.

The flow cells were cleaned prior to each sample injection by flushing for five minutes with tap water (approximately 3 bars of pressure) while submerged in an ultrasonic bath. The flow cells were then flushed with 20 ml filter sterilized (0.22 μm) tap water. Finally, the flow cells were flushed twice with the actual particle suspension using a glass pipette. The remaining suspension in the flow-cell was used for analysis.

Each measurement of suspended particles was repeated seven times for single particle type suspensions and three times for mixed suspensions, in between which the above cleaning procedure was applied.

### Size distribution analysis

Size distributions of particle suspensions were analyzed on a Malvern Zetasizer Nano dynamic light scattering system. Size distributions of drinking water samples were analyzed using a Beckman Coulter Multisizer 4e Coulter Counter with appropriate apertures.

### Microbiological analyses

The total number of bacteria were counted, and their morphology was examined after the sample was filtered onto a 0.22 μm black polycarbonate filter and stained by 4′,6-diamidino-2-phenylindole (DAPI) (Life Technologies D1306). After filtering an amount of sample, yielding 20–400 cells per 100 × 100 μm filter area, through the filter, 25 μl of 1 mg/ml DAPI was applied on top of the filter along with 1 ml of filter-sterilized (0.22 μm) tap water. After 15 min in the dark, the staining solution was sucked through the filter, which was then rinsed with filter-sterilized tap water (3 × 1 ml). Filters were mounted in immersion oil (Citifluor AF87) and examined by epi-fluorescence microscopy using a Zeiss Axio Imager 2 microscope. Plate counts at 22 and 37 °C was made according to ISO 6222:1999^31^ on yeast extract agar.

### Abiotic particle suspensions

10 g blue clay was taken from a freshly exposed surface of commercially available decoration clay and suspended in 1 l 0.2 μm filtered Milli-Q water. Size distribution analysis showed particle size clusters from 200 nm to 2 μm.

Ferroxyhyte and lepicocrocite were prepared according to Schwertmann and Cornell^32^ with the following precautions taken to avoid bacteria in the final suspensions: For ferroxyhyte, 300 ml 0.1M FeCl_2_·4H_2_O (Sigma Aldrich 44939) was filtered through a 0.22 μm PES filter and placed in a beaker. 5M NaOH (Merch 1.06498) was added dropwise while stirring until the pH reached 8. 40 ml 30% H_2_O_2_ (Merch 1.07209) was added rapidly, and the pH raised to 8 by adding 5M NaOH. The precipitate was centrifuged, washed and dried. Scanning electron microscopy showed mainly amorphous particles. For lepidocrocite, 11.93 g FeCl_2_·4H_2_O was dissolved in 300 ml Milli-Q water and filtered through a 0.22 μm PES filter. pH was adjusted to 6.7–6.9 with 1M NaOH and the solution aerated with 100 ml/min of air for 2–3 hours. The pH of the solution was constantly adjusted to remove protons produced by the oxidation process, adding approximately 120 ml NaOH. The precipitate was centrifuged, washed and dried. Scanning electron microscopy showed solid particles with sharp contours. Size distribution analysis showed a range from 200 nm to 10 μm with a maximum intensity at 600 nm.

10 g humic acid (Sigma Aldrich 53680) was dissolved in 1 liter 0.2 μm PES filtered 0.1 mM pyrophosphate, Na_4_P_2_O_7_ · 10H_2_O (Alfa Aesar A16195) (pH 9–10) based on Milli-Q water. The solution was placed on a magnetic stirrer for 24 hours and left to settle. The dissolved fraction was decanted and filtered through a 1.6 μm filter to remove remaining undissolved humic acid. The pH of the filtered solution was adjusted to below 6 with phosphorous acid (Prolab 20624.295), causing humic acid particles to precipitate. Size distribution analysis showed two major fractions around 150–400 nm and 6 μm.

All abiotic particle stock suspensions were preserved by 2% formaldehyde (AppliChem A0421), and checked for the presence of bacteria by DAPI staining and epi-fluorescence microscopy. Only few bacteria were detected in the blue clay stock. It was not possible to determine the number of cells by the DAPI staining method due to the low number compared to clay particles. It was estimated that bacteria constituted less than 0.1‰ of the total particles.

### Bacteria suspensions

Pure cultures of freeze dried *Lactococcus lactis* subsp. *lactis* (DSM no 20481), *Bacillus subtilis* (DSM no. 618), *Escherichia coli* (DSM no. 5695), and *Vibrio natriegens* (DSM no. 759) were obtained from Leibniz-Institut DSMZ-Deutsche Sammlung von Mikroorganismen und Zellkulturen GmbH. Cultures were revived according to the specifications given by DSMZ, grown in specific growth media. *L. lactis* was grown in 10 g peptone (Fluka 70172), 5 g yeast extract (Sigma Aldrich Y0500), 5 g glucose (Sigma G7528), and 5 g NaCl (Merck 1.06404) per liter. *B. subtilis* was grown in 5 g peptone and 3 g meat extract (Fluka 70164) per liter. *E. coli* was grown in 5 g peptone, 3 g meat extract, 800 mg NaCl, and 1.23 g MgSO_4_ (Fluka 00627) per liter. *V. natriegens* was grown in 5 g peptone, 3 g meat extract, and 15 g NaCl per liter. For solid growth media, 15 g/l agar (Merck 1.01614) was added (11 g/l for *E. coli*). All were incubated at 22 °C for 20 hours.

Unidentified bacteria were isolated from various Danish drinking water supplies by spreading and incubation on plate count agar (Merch 1.05463) plates at 22 °C for 20 hours and repeatedly transferring single colonies to new plates.

The purity of the monocultures was tested by DAPI staining and examination of morphology by epi-fluorescence microscopy. The size of individual cells ranged from 600 nm to 3 μm (longest axis), as determined by microscopy.

The cultures were stored at −80 °C in growth media with 30% (v/v) glycerol. Thawed cultures were incubated on appropriate plates (described above) for 20 hours, transferred to new plates, and incubated for an additional 20 hours. A single colony was transferred to 200 ml 0.22 μm filter-sterilized growth medium. The bacteria were allowed to grow for 20–40 hours depending on their growth rates and re-inoculated in 0.22 μm filtered media twice before they were used in measurements.

### Mixed suspensions

Prior to each mixed suspension measurements, the individual suspensions were measured in triplicate by the sensor to determine their concentration and mixed accordingly to achieve the desired ratio between bacteria and abiotic particles.

### Comparison between sensor response and total counts

A series of tap water aliquots were spiked with increasing concentrations of yeast extract (Sigma Aldrich Y0500): 0, 0.02, 0.2, and 2 mg/l. The bacteria concentration in the aliquots were measured by the bacteria sensor (3–6 repetitions) and by DAPI staining (20 randomly selected fields of view) at initiation and after 2, 7, and 11 days of incubation at room temperature.

### Particle size standards

Polybead polystyrene microspheres (2.5% solids latex) were obtained from PolyScience Inc. Warrington, PA, USA in the sizes 3.004 μm (17134, lot no. 629888), 1.53 μm (17133, lot no. 613529), 0.99 μm (08226, lot no. 644236), and 0.771 μm (07309, lot no.623715). Uniform, non-porous silica microspheres (SS03N, lot no. 8933) with a diameter of 690 nm were obtained from Bangs Laboratories, Inc., Fishers, IN, USA. Prior to dilution, stock suspensions were sonicated for 1–10 minutes to break any aggregation.

### Turbidity measurements

For comparison, a Hach Lange ULTRATURB sc turbidimeter was installed alongside a bacteria sensor at the inlet/outlet of a major closed drinking water reservoir in a large Danish non-chlorinated drinking water distribution system.

### Field test at a slaughterhouse

Around the time of an expected daily peak in bacteria and particle concentrations, samples were taken for laboratory analyses at intervals corresponding to the resolution of the sensor (approximately ten minutes). Samples were taken during flushing of the flow-cell and thus corresponded to the subsequent sensor response.

Single analyses of plate counts and total counts by DAPI staining were performed. The standard deviation of 10 counts on each stained filter was approximately 20%. Samples were homogenized by repeatedly passing through a syringe needle to break cell clustering and biofilm prior to analysis. However, a few clustered cells could still be observed within the stained samples.

## Additional Information

**How to cite this article**: Højris, B. *et al*. A novel, optical, on-line bacteria sensor for monitoring drinking water quality. *Sci. Rep*. **6**, 23935; doi: 10.1038/srep23935 (2016).
